# Dual Role of Cancer Epithelial-Specific TRAF3 in Regulating Breast Cancer Cell Survival and Lymphocyte Activity

**DOI:** 10.3390/ijms27104414

**Published:** 2026-05-15

**Authors:** Chaido Sirinian, Anne-Lise de Lastic, Harry Zaverdas, Martha Nifora, Dimitra Georgakopoulou, Martina Samiotaki, Maria Ioanna Argentou, Stavros Peroukidis, Søren E. Degn, Maria Rusan, Konstantinos Theofilatos, Seferina Mavroudi, Anastasios D. Papanastasiou, Angelos Koutras

**Affiliations:** 1 Molecular Oncology Laboratory, Division of Oncology, Department of Medicine, University of Patras, 26504 Patras, Greece; 2 Laboratory of Immunohematology, Department of Internal Medicine, Medical School, University of Patras, 26504 Patras, Greece; delastic@upatras.gr (A.-L.d.L.); up1019151@upnet.gr (D.G.); 3 Department of Nursing, School of Health Rehabilitation Sciences, University of Patras, 26504 Patras, Greece; h.zaverdas@insybio.com (H.Z.); smavroudi@upatras.gr (S.M.); 4 Department of Biomedical Sciences, University of West Attica, 12243 Athens, Greece; mnifora@uniwa.gr (M.N.); apapanasta@uniwa.gr (A.D.P.); 5 Protein Chemistry Facility, Biomedical Sciences Research Center “Alexander Fleming”, 16672 Athens, Greece; samiotaki@fleming.gr; 6 Breast Unit, Department of Surgery, University Hospital of Patras, 26504 Patras, Greece; marianna.argentou@gmail.com; 7 Panarkadikon General Hospital, 22100 Tripolis, Greece; st.peroukidis@gmail.com; 8 Department of Biomedicine, Aarhus University, 8000 Aarhus, Denmark; sdegn@biomed.au.dk; 9 Department of Molecular Medicine, Aarhus University Hospital, 8200 Aarhus, Denmark; marrus@rm.dk; 10 Department of Clinical Pharmacology, Aarhus University Hospital, 8200 Aarhus, Denmark; 11 Department of Clinical Medicine, Aarhus University, 8200 Aarhus, Denmark; 12 School of Cardiovascular and Metabolic Medicine & Sciences, King’s College London, London SE1 7EH, UK; k.theofilatos@insybio.com; 13 InSyBio PC, 26504 Patras, Greece

**Keywords:** breast cancer, TRAF3, cell survival, apoptosis, cancer immunity, tumor microenvironment

## Abstract

TRAF3 (TNF Receptor Associated Factor 3) is a regulator of NF-κB signaling, acting mainly as an inhibitor of the alternative NF-κB pathway. While TRAF3 has a well-established role in immune function, mainly via B- and T-lymphocyte regulation, its roles in cancer remain unclear. Breast cancer is the most common malignancy in women and a neoplasm displaying high levels of intratumoral heterogeneity. Identifying and understanding key molecules at the interface of breast cancer cells and the immune system is crucial for advancing therapeutic strategies for breast cancer patients. Here, by employing publicly available breast cancer datasets, breast cancer cell lines stably expressing TRAF3, mass spectrometry analysis in combination with functional assays, co-culture systems, and signal pathway characterization, we sought to assess the specific role of TRAF3 in breast cancer cells and how TRAF3-expressing breast cancer cells affect their immune microenvironment. Our results indicate that TRAF3 protein overexpression inhibits colony formation through apoptosis regulation. Proteome analysis for TRAF3 interactors and over-representation analysis identified multiple protein complexes related to cell cycle, apoptosis, and immune responses. Furthermore, TRAF3-expressing breast cancer cells displayed reduced levels of PD-L1 and when co-cultured with PBMCs induced a pro-inflammatory profile with increased CD16-NK cells and higher levels of IFN-γ and TNF-α and lower IL-10 and Tregs in the culture. These findings further expand the role of TRAF3 in breast cancer, not only as a regulator of EMT and survival of cancer cells, but also as a modulator of the tumor-immune microenvironment.

## 1. Introduction

Multiple pathways have been implicated in breast cancer initiation, progression and metastasis, such as PIK3CA-AKT, HER family signaling, MAP kinases and recently the Nuclear Factor-Kappa Beta (NF-κB) pathway [1,2,3]. The roles of these pathways in disease biology are divergent, highly heterogeneous, cell- and context-dependent and, at times, redundant. The NF- κB pathway has well-characterized roles both in health and disease [4,5]. The NF-κB pathway can be divided into two major activation modules: the classical (canonical) and the alternative (non-canonical) that often overlap in time and space [6]. In mammary carcinomas, NF- κB pathway activation has been identified mostly in HER2-positive and triple negative (ER-, PR-, HER2-) disease [7,8]. However, because of breast cancer heterogeneity and the complexity of the NF-κB pathway, the exact role of the classical and alternative modules in breast cancer development, progression and metastasis remains to be elucidated [9,10,11]. Tumor necrosis factor receptor-associated factors (TRAFs) are a family of intracellular adaptor molecules that function downstream of multiple TNFR family members and other non-RTKs (Receptor Tyrosine Kinases) [12]. Seven TRAF proteins have been identified and characterized through common domain structure. After receptor activation, TRAFs function as adapter proteins with E3 ubiquitin ligase activity that mediate a plethora of cytoplasmic signaling cascades, with central roles in the biology of immune cells but also cancer [13]. Signaling pathways involving TRAFs are related to the activation of several kinases and transcription factor pathways such as mitogen-activated protein kinases (MAPKs), nuclear factor-κB (NF- κB), and interferon-regulatory factors (IRFs) [14,15]. Notably, TRAFs have the capacity to regulate canonical and alternative NF- κB signaling in a cell- and context-dependent manner. Generally, TRAF2, 5, and 6 are activators and/or enhancers of the canonical NF-κB signaling pathway, while TRAF3 acts mainly as an inhibitor of the alternative NF- κB pathway, through Nuclear Factor- κB-Kinase (NIK) degradation [16,17]. TRAF3 seems to be the only TRAF with an inhibitory effect on NF-κB signaling. It also has a reported nuclear role in B-cells through inhibition of CREB-mediated transcription [18]. Surprisingly, TRAF3 was recently identified as a regulatory factor in density-dependent proliferation of epithelial cells, through alternative NF-κB signaling inhibition, but also the activation of innate immune genes [19]. Recently, we were able to ascribe a role to TRAF3 in breast cancer through the interaction of RANK-c in ER-positive cells [20]. Here, we attempt to elucidate an epithelial-specific role of TRAF3 expressed by cancer cells, but also how breast cancer-expressing TRAF3 cells affect tumor microenvironmental cells, with emphasis on immune cells. We were able to show that TRAF3 correlated with better clinicopathological parameters in breast cancer, affecting tumor cell survival. In addition, TRAF3-expressing breast cancer cells expressed a tumor-inhibitory cytokine profile when co-cultured with normal human PBMCs (peripheral blood mononuclear cells), altering T-regulatory and NK cell populations in co-cultures.

## 2. Results

### 2.1. TRAF3 mRNA and Protein Correlate with Improved Prognosis in Breast Cancer

**Figure 1:**
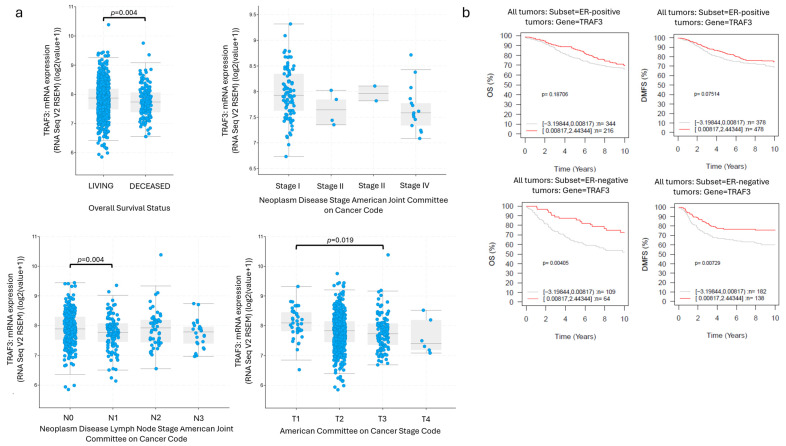
*TRAF3* is positively correlated with favorable prognosis in breast cancer. (**a**) High *TRAF3* mRNA expression levels are associated with better OS (Living vs. Diseased, Mann–Whitney U test), lower disease stage (Bonferroni correction), lower lymph node stage (N) (N0 vs. N1: *p* = 0.004, Bonferroni correction) and lower tumor stage (T) (T1 vs. T3: *p* = 0.019, Bonferroni correction) in the TCGA-BRCA cohort. (**b**) High *TRAF3* mRNA expression presents with a statistically significant better OS (*p* = 0.00405) and DMFS (*p* = 0.00729) in the ER-negative breast cancer cohort employed by GOBO, with ER-positive disease presenting a similar association despite not reaching statistical significance.

Our previous results have shown that *TRAF3* levels present a reverse correlation with histological grade, tumor diameter and proliferation index ki-67 while at the same time *TRAF3* expression at the mRNA level has a significantly positive impact on Overall Survival (OS), Recurrence Free Survival (RFS) and Distant Metastasis Free Survival (DMFS) [20]. In order to further elucidate the role of *TRAF3* in breast cancer, we analyzed TCGA BRCA through cBioPortal and GOBO for *TRAF3* expression in relevance to ER status and several clinicopathological parameters. High levels of *TRAF3* expression were correlated with Survival Status, lower lymph node stage (*p* = 0.002, Kruskal–Wallis test), lower tumor stage (*p* = 0.012, Kruskal–Wallis test) and a reverse association with neoplasm stage (*p* = 0.442, Kruskal–Wallis test) even if not statistically significant (Figure 1a). In addition, *TRAF3* expression presented a significant positive correlation with OS and DMFS of ER-negative breast cancer cases and a similar association with OS and DMFS of ER-positive (Figure 1b). Finally, when breast cancer cases were divided based on PAM50 molecular subtyping, *TRAF3* expression levels were positively correlated with OS and DMFS in Basal and HER2-enriched tumors (Supplementary Figure S1a,b). These results indicate that *TRAF3* expression in breast cancer associates with better patient prognosis and, especially in the ER-negative, Basal and HER2-enriched groups.

### 2.2. Breast Cancer Cell Enforced TRAF3 Expression Affects Cell Phenotype and Survival

**Figure 2:**
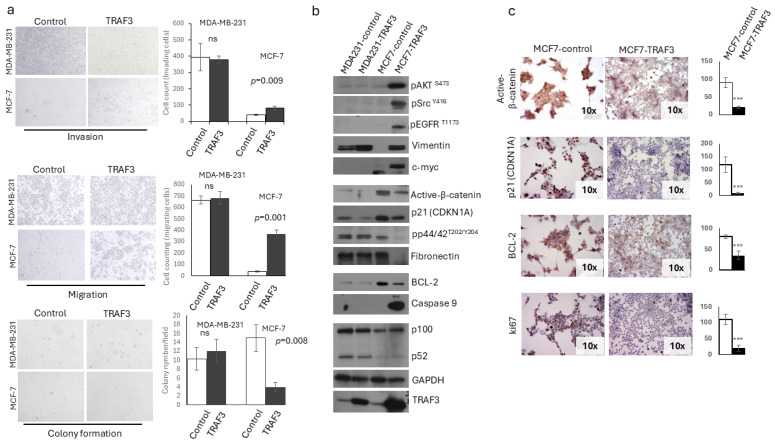
Forced TRAF3 expression in breast cancer cell lines induces partial EMT and affects cell proliferation. (**a**) Invasion, migration and colony formation assays depicting an opposing phenotype between migratory and proliferative states of MCF7-TRAF3 cells. (**b**) Western blot analyses for the indicated proteins in MDA-MB-231 and MCF-7 cells (control and TRAF3 expressing). (**c**) ICC for the indicated proteins in MCF-7 cells, indicating significant downregulation of key molecules upon TRAF3 expression (ns: no significance; *** *p* < 0.001 Student’s *t*-test).

With the aim of identifying epithelial-specific functions of TRAF3 in breast cancer cells, we stably transduced MCF-7 and MDA-MB-231 cells to express human *TRAF3*. While forced expression of *TRAF3* in MDA231-TRAF3 (MDA-MB-231-TRAF3) cells had minimal or no effects on cellular phenotype and aggressive properties, MCF-7 cells overexpressing TRAF3 had a pronounced change in their shape and ability to reach confluency, losing cohesiveness and acquiring a more mesenchymal phenotype, while at the same time enhancing their migratory and invasive capacity, while reducing their colony formation competence. In more detail, MCF7-TRAF3 (MCF-7-TRAF3) breast cancer cells attained a more spindle cell-like phenotype resembling an EMT, presenting with significantly enhanced capacity to invade matrigel substrate and altered active-beta-catenin, Vimentin and Fibronectin expression (Figure 2a,b and Supplementary Figure S2a). Western blot and immunocytochemistry (ICC) revealed a partial EMT ongoing process with activated AKT kinase, SRC kinase, p65 and EGFR receptor in MCF7-TRAF3 cells, but not in MDA231-TRAF3, and with no significant changes for TRAF2 and NFKB2 (p100) in both cell lines (Figure 2b,c and Supplementary Figure S2d). On the other hand, MCF7-TRAF3 cells presented a significantly diminished capacity to form colonies in a soft-agar anchorage-independent growth assay and had reduced expression of activated-beta-catenin, p21, phosphorylated ERK kinase and proliferation marker ki-67. This indicates a possible proliferation defect of TRAF3-expressing cells in contrast to MCF7-control cells and MDA-MB-231 (control and TRAF3) cells that presented with no significant differences (Figure 2b,c and Supplementary Figure S2b). Proliferation index ki-67 downregulation indicates that TRAF3-expressing cells exit the cell cycle, in accordance with a role of TRAF3 in regulating epithelial cell density [19]. To test if there was a proliferation arrest or an apoptosis activation, we stained MCF7-TRAF3 cells for BCL-2 and Caspase-9, two central molecules in the apoptotic cascade. MCF7-TRAF3, but not MDA231-TRAF3 cells, downregulated anti-apoptotic BCL-2 proteins and simultaneously upregulated Caspase-9, pointing to a possible TRAF3-dependent activation of the pro-apoptotic cascade (Figure 2b,c and Supplementary Figure S2b). Further, *TRAF3* mRNA levels were inversely correlated with BCL-2 protein expression in the TCGA BRCA cohort supporting our findings that TRAF3 expression can negatively affect BCL-2 (Supplementary Figure S2c). The above findings indicate that TRAF3 regulates cancer cell morphology and survival through partial EMT and apoptosis pathways, at least in ER-positive cancer cells.

### 2.3. Gene Enrichment Analysis and TRAF3 Interactome Identify Altered Cell Cycle and Immune-Related Pathways

**Figure 3:**
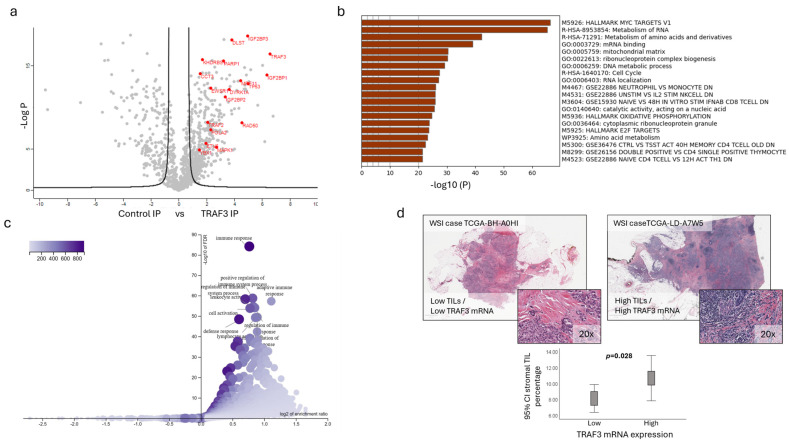
Identification of interactors, pathways and processes of TRAF3 in breast cancer. (**a**) Volcano plot of significant TRAF3 interactions in MCF-7 cells (FDR < 0.05). (**b**) Top 20 enriched pathways (Metascape) among proteins that interact with TRAF3 in MCF-7 cells with −log10(Padj) > 10^−20^. (**c**) Significantly enriched pathways among genes co-expressed with *TRAF3* in the TCGA BRCA cohort. (**d**) Representative BRCA cases from the TCGA cohort presenting with High and Low TILs (upper panel). High *TRAF3* mRNA expression is correlated (*p* = 0.02, Mann–Whitney U Test) with High stromal TILs in the TCGA BRCA cohort (*n* = 200).

To identify protein interactome-related alterations differentially affecting apoptosis activation and cell behavior in the two cell lines, we immunoprecipitated TRAF3 from MCF7-TRAF3 cells and subjected those affinity-enriched samples to mass spectrometry (LC-MS/MS) analysis. As expected, TRAF2, ETV6 and DYRK1A were amongst the 429 significantly enriched proteins in TRAF3 immunoprecipitated samples compared to the controls, confirming the previously established TRAF3/TRAF2, TRAF3/ETV6, and TRAF3/DYRK1A interactions [21] (Figure 3a). Over-Representation Analysis (ORA) on the 429 TRAF3 interactome partners from MS through METASCAPE [22] identified significantly (Padj < 10^−20^) enriched terms such as HALLMARK MYC TARGETS V1, mitochondrial matrix, cell cycle, GSE22886 NEUTROPHIL VS MONOCYTE DN, and GSE15930 NAIVE VS 48H IN VITRO STIM IFNAB CD8 TCELL DN (Figure 3b). Further, an ORA analysis through WebGestalt [23] on 6306 significantly co-expressed genes with *TRAF3* in the TCGA BRCA dataset (960 complete samples) identified multiple GO terms such as immune response (GO:0006955), positive regulation of immune system processes (GO:0002684), and adaptive immune response (GO:0002682) significantly (Padj < 10^−40^) associated with *TRAF3* expression in breast cancer (Figure 3c). In addition, we selected 200 cases from the TCGA BRCA cohort with a complete clinicopathological profile and high quality WSI’s (Whole Slide Images) and quantified stromal TIL (Tumor Infiltrating Lymphocytes) presence in relevance to *TRAF3* mRNA expression. *TRAF3* expression presented a significant positive correlation (*p* = 0.006) with the presence of lymphocytes (stromal TILs) at the tumor microenvironment (TME), and when cases were separated based on mean *TRAF3* expression as High and Low, TIL presence correlated (*p* = 0.028) with the *TRAF3* high expression group (Figure 3d). However, due to bulk tumor sequencing (RNA seq) of the TCGA samples, *TRAF3* expression cannot be attributed exclusively in the cancer epithelial cell compartment, nor can we exclude other TME cell-dependent contributions to *TRAF3* mRNA expression levels. This analysis further supports a possible role for TRAF3 in cell cycle and apoptosis regulation of breast cancer cells, while at the same time suggests a possible function of TRAF3 on the tumor-related immune response in breast cancer.

### 2.4. Single-Cell Transcriptomics Reveal TRAF3-Expressing Cancer Epithelial Cell Types in Breast Tumors

**Figure 4:**
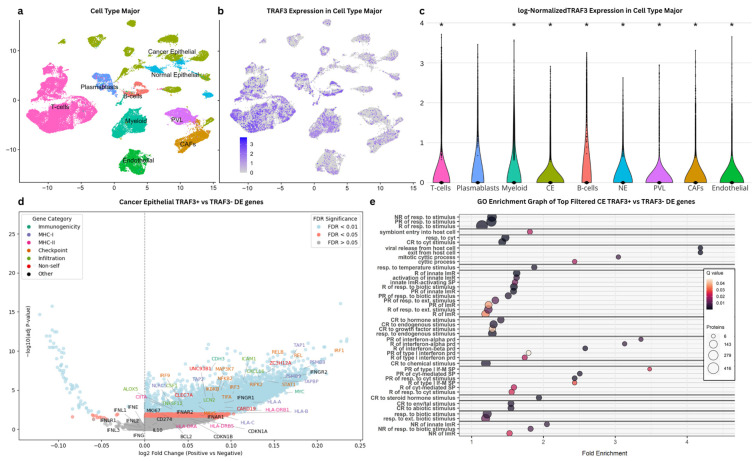
*TRAF3* expression across cell populations in the scRNA human breast cancer dataset. (**a**) UMAP visualization of 81,389 quality-filtered single cells derived from the Breast Cancer Atlas, colored by cell type annotation. (**b**) Feature plot showing log-normalized *TRAF3* expression projected onto the UMAP embedding. (**c**) Violin plots depicting log-normalized *TRAF3* expression across each of the cell types. Statistical comparisons were performed using Wilcoxon rank-sum tests, comparing each cell type against all remaining cells, followed by Benjamini–Hochberg correction for multiple testing. Asterisks (*) indicate adj *p*-values < 0.05. (**d**) Volcano Plot of Differential expression of *TRAF3*-positive (*TRAF3*+) vs. negative (*TRAF3*-) Cancer Epithelial (CE) cells. The x-axis represents the log_2_ fold change of expression in *TRAF3*-positive versus *TRAF3*-negative cells, and the y-axis shows the −log_10_ adjusted *p*-value (FDR). Points are colored according to FDR significance, while labels highlight specific immunologically relevant genes, colored according to the following categories: (i) Immunogenicity—Immunogenicity/Antigen Presentation; (ii) MHC-I—MHC class I pathway (CD8^+^ T-cell recognition); (iii) MHC-II—MHC class II (tumor-intrinsic or antigen-presenting cell mediated); (iv) Checkpoint—Checkpoint blockade/Immune Modulation; (v) Infiltration—Increase immune infiltration into tumors; and (vi) Non-self—Promote tumor cell recognition as “non-self”. Selected genes of interest not in the above categories are colored black (‘Other’ category). (**e**) Gene Ontology (GO) Enrichment Analysis of the filtered top DE genes (FDR < 0.05 & |log2FC| > 0.1) identified via differential expression analysis between *TRAF3*+ and *TRAF3*-cancer epithelial (CE) cells. X-axis represents the Fold Enrichment, and y-axis represents the immune-related Biological Process and Molecular Function GO terms, grouped into clusters based on functional similarity (for the full GO term graph with all the immune and non-immune related GO terms, see Supplementary Figure S3). Dot size is analogous to the number of specific genes associated with each GO term, while their color gradient corresponds to the FDR-adjusted *p*-value (Q value). Abbreviations used include the following: CE (Cancer Epithelial cells), NE (Normal Epithelial cells), PVL (PeriVascular-Like cells), CAFs (Cancer-Associated Fibroblasts), PR (Positive Regulation), R (Regulation), prd (production), MM (Molecular Mediator), MBP (Macromolecule Biosynthetic Process), MMP (Macromolecule Metabolic Process), CR (Cellular Response), env/tal (environmental), RSP (receptor signaling pathway), SP (signaling pathway), resp. (response), ext. (external), and If-M (interferon-mediated).

To explore a possible role of cancer cell-expressed *TRAF3* in regulating activation of infiltrating immune cells of the tumor microenvironment in breast cancer, we analyzed scRNA-seq data from a cohort of 26 primary breast tumours [24]. The data were obtained from the single-cell and spatially resolved atlas of human breast cancers published by Wu et al. [24] encompassing malignant epithelial cells as well as diverse immune, stromal, and endothelial cell populations. After quality control filtering, 81,389 high-quality cells were retained for downstream analysis. By utilizing dimensionality reduction with the major cell type annotations, these cells revealed a diverse cellular landscape comprising malignant epithelial cells, stromal populations, and various immune cell subsets (Figure 4a,c). *TRAF3* was found to be expressed across multiple cell types within the tumor microenvironment, including both malignant and non-malignant populations (Figure 4b), with a significant expression in cancer epithelial (CE) cells compared to the rest of the cell types combined. To define pathways and mechanisms associated with *TRAF3* expression in the cancer epithelial (CE) cells, these were stratified into *TRAF3*-positive (*TRAF3*+, expression > 0) and *TRAF3*-negative (*TRAF3*-, expression = 0) populations. It is important to note that zero values in scRNA-seq may also reflect technical dropout or expression below the detection threshold, and this comparison should be interpreted cautiously. Differential expression analysis using these two groups identified a wide set of genes, the expression of which was significantly associated (FDR < 0.01) with *TRAF3* expression (Figure 4d). The majority of significantly differentially expressed immune-related genes exhibited higher expression in *TRAF3*-positive CE cells relative to *TRAF3*-negative cells. Specifically, many of the MHC-I pathway genes (HLA-A, HLA-B, HLA-C, TAP1, TAP2, TAPBP, PSMB8, PSMB9, NLRC5) and checkpoint/immune modulation genes (STAT1, IRF1, IRF3, IRF9, REL, RELB, NFKB2, IKBKB, MAP3K7, MAVS, RIPK2, TIFA) clustered at the upper-right quadrant, with high statistical significance (adj *p*-Value) and high log2FC. This indicated that *TRAF3*-positive CE cells exhibit an upregulation of genes involved in antigen presentation and immune checkpoint pathways, potentially having a role in enhancing immunogenicity and response to immune surveillance. To further investigate the immunological pathways and mechanisms related to *TRAF3* expression in CE cells, a Gene Ontology (GO) Enrichment Analysis on selected DE genes was performed. Specifically, for the filtering of the DE genes, we used FDR < 0.05 with |log2FC| > 0.1 (which lies at the lower boundary of the effect-size distribution) to remove biologically trivial changes near zero, while retaining the majority of modest but potentially meaningful DE genes. In the retained set, this analysis revealed a pronounced enrichment for immune-related biological processes and molecular functions (Figure 4e and Supplementary Figure S3). In particular, enrichment clustering identified 14 high-scoring clusters, with several dominated by immune- and stimulus-associated GO terms showing low q-values (<0.01) and high fold enrichment. Among the most prominent terms was the regulation of innate immune response, activation of innate immune response, positive regulation of innate immune response, and regulation of response to biotic stimulus (all FE~1.6, q < 0.01). Other terms highlighted interferon-related processes, including positive regulation of interferon-α production (FE~3.4, q < 0.01), regulation of interferon-α production (FE~3.1, q < 0.01), and regulation of interferon-β production (FE~2.6, q < 0.01). Further emphasis was given to cytokine signaling, with positive regulation of cytokine-mediated signaling pathway and positive regulation of response to cytokine stimulus (both FE~2.5, q < 0.01), while host–pathogen processes were also reported, such as viral release from host cell and exit from host cell (FE~4.2, q < 0.01). This supports the case that high *TRAF3* expression in CE is associated with a transcriptional program enhancing immune surveillance. It is worth noting that these immune-associated transcriptional programs were detected within malignant epithelial cells (CE), indicating that *TRAF3* expression may characterize subsets of cancer cells with immune-interactive or immunomodulatory transcriptional states rather than reflecting immune cell contamination. However, for the reasons mentioned before, the results of this single-cell analysis should be considered supportive rather than definitive. But nevertheless, these findings, together with the identification of multiple upregulated immune-relevant genes, support a model in which *TRAF3* expression in CE is linked to tumor–immune interactions in the TME.

### 2.5. Breast Cancer Cell-Specific TRAF3 Affects Lymphocyte Cell Populations and IFN Gamma Production

**Figure 5:**
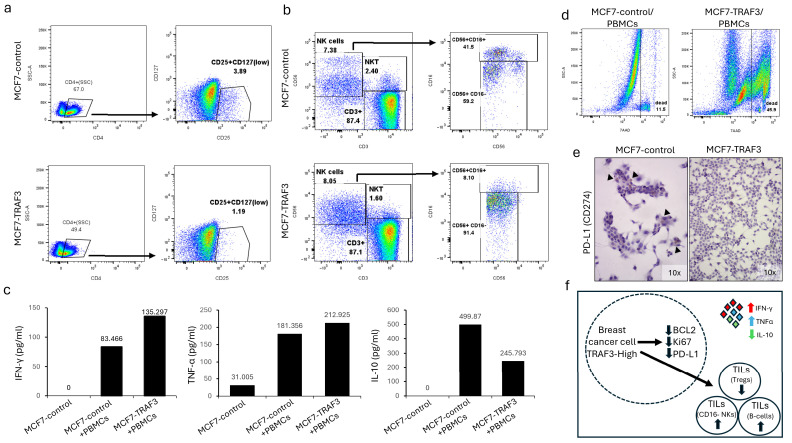
TRAF3 expression in cancer cells affects PBMC subpopulations and cytokine expression. (**a**) FACs analysis of PBMCs co-cultured with MCF7-TRAF3 cells indicates the downregulation of the CD25+CD127low (Tregs) subpopulation of CD4+ T cells. (**b**) FACS analysis of PBMCs co-cultured with MCF7-TRAF3 cells indicates the upregulation of the CD56+CD16- subpopulation of NK-cells. (**c**) Diagrams depicting absolute quantification of IFN-γ, TNF-α and IL-10 in the supernatants of co-cultured PBMCs/MCF7-TRAF3 cells. (**d**) FACs analysis for live/dead MCF-7 breast cancer cells co-cultured with PBMCs depicting a shift from alive to dead cells in the MCF7-TRAF3 cell population in comparison to MCF7-control cells. (**e**) IHC stain for PD-L1 (CD274) on MCF7-control and MCF7-TRAF3. Arrowheads depict PD-L1 expression only on MCF7-control cells. (**f**) Schematic illustration of a proposed model of TRAF3 action in breast cancer epithelial cells and on the surrounding tumor microenvironmental cells.

With the aim to test a possible cellular interaction between TRAF3-expressing breast cancer cells and immune cells, we employed MCF7-TRAF3, MDA231-TRAF3 and normal peripheral blood mononuclear cells (PBMCs) in a co-culture system for 3 days. Freshly isolated, normal donor PBMCs were placed in culture medium for three days or co-cultured with MCF7-TRAF3, MDA231-TRAF3 and relevant mock transduced counterparts. Furthermore, cell supernatants from each experimental condition were collected for cytokine analysis at the end of culture in order to identify changes in interferon and interleukin concentrations due to cancer–immune cell interactions. PBMC activation was already evident on the second day of co-culture by aggregate formation on cancer cells, in all culture systems employed (Supplementary Figure S4a). Through flow cytometric analysis of the surface markers of the PBMCs, a reduction was identified on T regulatory lymphocytes (Tregs), characterized by the phenotype CD4+CD25+CD127low, in the MCF7-TRAF3 culture in comparison with its control (1.19% vs 3.89% Tregs on CD4+, respectively) (Figure 5a and Supplementary Figure S4b). In addition, there was a marked expansion of the CD56+CD16- subpopulation of NK cells in the same experimental conditions (91.4% vs. 59.2% on total CD56+ cells) (Figure 5b and Supplementary Figure S4c) accompanied by a modest reduction of NKT cells. At the same time, we observed an upregulation of the CD20+ B lymphocytes in the co-cultured PBMCs in TRAF3-expressing cell lines in comparison to control culture systems, indicating a possible survival benefit for B-cells when interacting with TRAF3-expressing cancer cells (Supplementary Table S2). There were no observed differences in the relative percentages of CD4 and CD8 lymphocytes and their expression of the activation markers CD69 and HLA-DR (Supplementary Table S2). When supernatants of the co-cultures were analyzed for cytokine production, MCF7-TRAF3/PBMCs displayed increased levels of IFN-γ and TNF-α with concomitant IL-10 reduction compared to their counterpart (Figure 5c and Supplementary Table S3). To assess the functional significance of these findings on breast cancer cells, we performed a viability study (by incorporation of a 7-AAD analog) on all cell lines at the end of their co-culture with PBMCs, where both MDA231-TRAF3 and MCF7-TRAF3 displayed increased apoptosis compared to their respective controls (Figure 5d and Supplementary Figure S5a). Notably, after TRAF3-expressing MCF-7 cells were co-cultured with PBMCs, 45.9% were dead vs. only 11.5% for MCF-7 that did not express TRAF3, comparable to MCF-7 cells without PBMCs which presented with 13.4% dead cells (Supplementary Figure S5b). Finally, we observed through ICC the downregulation of PD-L1 protein (*CD274*) on MCF-7 and MDA-MB-231 cells expressing TRAF3 (Figure 5e and Supplementary Figure S5c). This finding was also confirmed in single-cell data, where *TRAF3* mRNA expression in cancer epithelial cells presented a reverse correlation with *CD274* (PD-L1) mRNA expression, but also in breast cancer cell lines from the Cancer Cell Line Encyclopedia through cBioPortal, where TRAF3 protein expression presented with an inverse correlation with CD274 (PD-L1) protein expression (Supplementary Figure S5d,e). Collectively our results indicate that TRAF3-expressing MCF-7 cells downregulate PD-L1 and induce a pro-inflammatory profile on the immune cells in their vicinity, supporting a role for TRAF3 in the immunogenicity of cancer epithelial cells (Figure 5f).

## 3. Discussion

Breast cancer is the most common malignancy amongst women, with immense levels of heterogeneity at the molecular and histological levels [25]. Multiple pathways and molecules regulate disease initiation, progression and metastasis, with NF-κB playing important roles in disease biology [26]. TRAFs are adaptor proteins that regulate NF-κB signaling and their overexpression in breast cancer leads to worse patient outcomes [27,28]. TRAF3 is the only inhibitory TRAF molecule on NF-κB signaling, and our previous work indicates that *TRAF3* expression correlates with reduced tumor size, lower histological grade and lower levels of proliferation index ki-67 [20]. We re-evaluated our findings in multiple publicly available breast cancer datasets through cBioPortal and GOBO, and at the mRNA level, we were able to show that higher expression of *TRAF3* correlated with better OS, RFS, lymph node stage (N) and tumor stage (T). Next, we sought to identify the functional effects of *TRAF3* forced expression in MCF-7 and MDA-MB-231 breast cancer cell lines. TRAF3 expression affected mostly MCF-7 cells, inducing an EMT-like phenotype, but with reduced capacity to form colonies in a clonogenic assay and reduced proliferation activity as judged by ki-67 stain. At the same time, the phenotype of TRAF3-overexpressing MCF-7 cells in culture seemed to follow the findings of Fomicheva and Macara, where epithelial TRAF3 regulates density-dependent cell proliferation [19]. In line with this report, MCF7-TRAF3 cells had minimal ki-67 expression, suggesting a G0 exit, and were unable to reach high confluency. Further, TRAF3 in MCF-7 cells downregulated BCL-2, a well-established anti-apoptotic molecule with significant roles in multiple cancer progression models, and upregulated Caspase-9, a marker of apoptosis [29,30]. While TRAF3 expression seems to affect mostly ER-positive cells (MCF-7) in culture, patient data reveal a stronger dependence of ER-negative breast cancer on TRAF3 expression. This finding can be attributed to the high breast cancer heterogeneity as a disease, the involvement of more than one cell type that contributes to TRAF3 expression levels in patient samples and the complex cell- and context-specific function of TRAF3 in breast.

Cellular protein functions are largely determined by protein–protein interactions that are tightly regulated in a cell- and context-dependent manner [31,32]. In that way, we immunoprecipitated TRAF3 from MCF-7 cells to identify interactors in breast cancer cells that determine functional outputs. We found multiple protein molecules with divergent roles, and enrichment analysis for these interactors points to several pathways and functional modules, such as proliferation regulation, cell cycle and immune cell regulation. Similar results were retrieved when we analyzed genes significantly co-expressed with TRAF3 in the TCGA BRCA dataset, through Over-Representation Analysis. The finding that *TRAF3* expression correlated with immune cell signatures was also corroborated by TIL analysis in the TCGA BRCA cohort, which showed a positive association of TILs with *TRAF3* mRNA expression. While TRAF3 affects cell morphology and proliferation of breast cancer cells, our analysis indicates a possible role for TRAF3 in the immunogenicity of the tumor cells and in their capacity to interact with cells of the immune system. Breast cancer single cell analysis showed that TRAF3 is expressed in multiple cell types in a breast tumor and in cancer epithelial cells, amongst others. Single-cell transcriptomics, in general, has emerged as a useful approach for resolving tumor cell states, intratumoral heterogeneity, and immune-interactive programs within complex tumor ecosystems, although the interpretation of these data remains context-dependent and subject to technical limitations such as sparse expression and dropout [33]. This consideration further prompted us to study the interaction between breast cancer cells and cells of the TME. It is important to acknowledge that the single-cell analysis should be interpreted with caution. Our aim was not to define global transcriptional differences between normal and malignant epithelial cells, but to explore phenotypic changes associated with TRAF3-positive versus TRAF3-negative cancer epithelial states, thus differentiating CE cells between TRAF3 > 0 versus TRAF3 = 0 expression states, while revising the analysis to account for patient-of-origin and epithelial subtype composition. However, some TRAF3-undetected cells may reflect technical dropout or expression below the detection threshold rather than true biological absence. Therefore, we consider the single-cell findings supportive of the broader conclusions of the study rather than definitive on their own.

This finding further prompted us to study the interaction between breast cancer cells and cells of the TME. Indeed, when TRAF3-expressing MCF-7 cells were co-cultured with normal human PBMCs, there seemed to be a polarization of the immune profile of the culture towards a pro-inflammatory response, which could be mediated through the expanded CD16- NK sub-population. This NK subset that normally comprises about 10% of the total NK population in the peripheral blood [34] has enhanced capability of IFN-γ production [35] (of which there were indeed higher levels in the MCF7-TRAF3 co-culture vs its control) and has been shown to impart a favorable prognosis when found in the tumor microenvironment [36]. Increased levels of IFN-γ and ΤNF-α are directly cytotoxic to tumor cells [37,38] and indirectly promote a highly inflammatory milieu, increasing adhesion molecules and chemokine production, which supports immune cell recruitment [39,40]. Moreover, one of the main caveats of IFN-γ and TNF-α action, namely the induction of PD-L1 in cancer cells via JAK/STAT1/IRF1 signaling leading to increased PD-L1 expression and adaptive resistance to T-cell and NK-cell attack, seems to be defective or inactive in our model of TRAF3 expression, where PD-L1 is downregulated. In accordance with the described profile of the MCF7-TRAF3/PBMC co-culture, the subset of regulatory T lymphocytes (Tregs) that represents one of the main immunosuppressive subpopulations in the TME [41] appears to be diminished, along with related cytokines such as IL-10. These findings lead to a possible susceptibility of TRAF3-expressing cancer cells to immune mediated death, involving the CD16-NK subset, in a context of impaired cancer-related tolerogenic mechanisms such as the induction of a suppressive microenvironment through Treg induction or PD-L1 mediated anergy. The above data indicate that TRAF3 can affect breast cancer initiation and progression through multiple molecular and cellular pathways, involving both cancer cell intrinsic mechanisms, but also through indirect regulation of the TME and specifically immune cells by TRAF3-expressing cancer cells. Nevertheless, our work has several limitations. Recent data indicate an inhibitory role of TRAF3 in MHC-I and PD-L1 expression in melanoma and neuroblastoma cells, respectively [42,43]. These data, even if acquired in non-epithelial, non-breast carcinoma systems, indicate the biologic complexity of TRAF3 function and a possible cell- and context-specific mechanism of action, which is also observed in our MDA-MB-231 system, a unique triple-negative, fibroblastic-like, k-Ras driven breast cell line, not affected by TRAF3 overexpression. The complexity of breast cancer as a disease and the cell- and context-dependent role of TRAF3 requires in-depth analysis and identification of the exact molecular pathways that underlie the regulation of immune cells through the interaction with TRAF3-expressing epithelial cells in the TME. These considerations are also relevant for the scRNA-seq analysis, where patient heterogeneity and dropout-related undetected genes, warrant cautious interpretation about the distinction in TRAF3-detected vs -undetected states.

## 4. Materials and Methods

### 4.1. mRNA Expression Dataset Analyses

Publicly available mRNA expression data was analyzed and visualized in breast cancer cohorts through cBioPortal [44,45] (TCGA-BRCA) and the GOBO on-line tool [46] (Chi, GSE11121, GSE12093, GSE2034, GSE2603, GSE5327, GSE6532, GSE7390, GSE12093, GSE1456, GSE3494, GSE7390) presented in detail in Supplementary Materials and Methods.

### 4.2. Cell Cultures, Plasmids and Antibodies

Breast cancer cell lines MDA-MB-231 and MCF-7 were purchased from ATCC (Manassas, VA, USA) and cultured in 37 °C, 5% CO_2_, in DMEM medium with 10% FBS and pen/strep. The TRAF3 coding sequence was amplified from a pCMV6-TRAF3-GFP (RG210417, Origene, Rockville, MD, USA) vector and cloned into pLenti-EF1a-GFP-2A-Puro (LV067, ABM Inc, Richmond, BC, Canada). The MDA231-TRAF3 and MCF7-TRAF3 stable cell lines and respective control cells were generated by transduction with a 2nd Generation lentivirus packaging plasmid mix (LV003, ABM Inc.), and positive clones were selected with puromycin. The antibodies employed in this study were the following: Phospho-AKT (#4060 1:2000, CST, Danvers, MA, USA), phospho-src (#6943, 1:1000, CST), phospho-EGFR (AF3394, 1:200, R&D systems, Minneapolis, MN, USA), Vimentin (#5741, 1:1000 CST), c-myc (sc-40, 1:200, SCBT, Paso Robles, CA, USA), active β-catenin (05-665, 1:500, Millipore, Darmstadt, Germany), p21 (#2947, 1:1000, CST), BCL-2 (sc-7382, 1:200, SCBT), caspase-9 (sc-7885, 1:200, SCBT), GAPDH (sc-25778, 1:1000, SCBT), Fibronectin (sc-52331; 1:200, SCBT), phospho-NF-κB p65 (#3033; 1:1000, CST), NF- κB2 p100/p52 (#3017; 1:1000, CST), phospho-p44/42 (ERK1/2) (#4376; 1:1000, CST), TRAF3 (#33640, 1:2000, CST), TRAF2 (sc-136999; 1:200, SCBT), and phospho-IkBa (#9246; 1:1000, CST) for WB and ki-67 (MIB-1, RTU, Biocare, Pacheco, CA, USA), BCL-2 (sc-7382, 1:60, SCBT), p21 (sc-817, 1:50, SCBT), active β-catenin (05-665, 1:50, Millipore), and PD-L1 (M3653, 1:60, DAKO, Glostrup, Denmark) for ICC.

### 4.3. Colony Formation, Migration and Invasion Assays, Immunoblotting and Immunocytochemistry

In order to measure the colony formation, migration and invasion capability of MDA231-TRAF3 and MCF7-TRAF3 cells and perform western blots and immunocytochemistry, we followed the relevant procedures as previously reported [20] and presented them in more detail in Supplementary Materials and Methods. For colony formation, cells (2.5 × 10^4^ cells/well) were suspended in 1 mL of 10% FBS DMEM containing 0.5% agarose and plated on a semisolid medium (DMEM with 10% FBS and 0.7% agarose) in a 12-well plate. Cells were then placed in a 37 °C and 5%CO_2_ incubator. The next day, 500 μL of 10% FBS DMEM was added to each well and changed every three days for 12 days. Images were obtained using an inverted microscope (Axiovert 40 CFL, AxioCam ERc, Carl Zeiss Microscopy GmbH, Oberkochen, Germany). All experiments were done in triplicates. For the migration assay (Transwell chambers, Corning Inc. NY, USA) and invasion assay (Matrigel, Corning Inc.), on the day of the experiment, culture chambers were incubated with serum-free medium for 2 h in a 37 °C/5%CO_2_ incubator. Totals of 2 × 10^5^ cells/chamber (migration) and 2 × 10^4^ cells/chamber (invasion) were resuspended in serum-free medium and seeded in the upper chamber of the assay and left to migrate (12 h) or invade (24 h) towards full medium (DMEM, 10% FBS). For counting migrating and invading cells, the chamber’s membrane was washed with 1xPBS, fixed for 10 min with 4% PFA and for 20 min with methanol, and stained with Giemsa for 5 min. Non-migrating or non-invasive cells were removed with a cotton swab. Images were obtained using an inverted microscope (Axiovert 40 CFL, AxioCam ERc, Zeiss). Each experimental procedure was repeated at least three times (biological or technical replicates, as required).

### 4.4. Sample Preparation and Mass Spectrometry Analysis

Cells were lysed in a 0.5% NP-40 lysis buffer (150 mM NaCl, 20 mM HEPES, 0.5 mM EDTA, 1 mM Na_3_VO_4_, proteinase inhibitor cocktail (Calbiochem, Darmstadt, Germany)). Cell extracts from each condition were incubated overnight with 6 μg of TRAF3 antibody (33640, CST) at 4 °C and, the next day, retrieved with 20 μL of Dynabeads protein G (Invitrogen, Carlsbad, CA, USA) for 2 h at 4 °C. Immunoprecipitates were washed three times in lysis buffer to remove unbound proteins. Beads were washed three times with 20 mM Hepes buffer pH 7.5 and resuspended in 1% SDC buffer (1% sodium deoxycholate in 100mM Tris pH 8.5). LC-MS/MS was performed using the NanoElute2 liquid chromatography system coupled to a timsTOF Ultra2 massspectrometer (Bruker Daltonik, Bremen, Germany), and the data were analyzed in Spectronaut 19.5 and Perseus (version 1.6.15.0) [47]. Student’s *t*-tests for independent variables were performed for each comparison taken into account in this study. *p*-values were adjusted for multiple testing using a permutation-based false discovery rate (FDR) correction with 250 randomizations. Differential expression results were visualized as heatmaps and volcano plots using Perseus built-in functions. MS analysis was performed on technical triplicates.

### 4.5. TIL Quantification on WSI from the TCGA BRCA Dataset

A dataset of 200 cases (Supplementary Table S1) of invasive breast carcinoma of no special type (IBC-NST) from the TCGA-BRCA cohort was compiled. For each case, the corresponding diagnostic H&E whole-slide image (WSI) was downloaded and evaluated for tumor-infiltrating lymphocytes (TILs). Stromal and intratumoral TIL scoring was performed according to the recommendations of the International TILs Working Group [48]. All assessments were performed by two pathologists with breast carcinoma experience. In addition, the presence/absence of tertiary lymphoid structures (TLS) and the presence/absence of increased TILs at the invasive tumor border were recorded. Stromal TIL assessment was frequently heterogeneous across the tumor stroma; in such cases, a weighted stromal TIL percentage was calculated by combining regional TIL estimates according to their approximate area contribution.

### 4.6. PBMCs Isolation and Co-Culture with Breast Cancer Cells

Whole blood was diluted 1:1 with DMEM, and PBMCs were isolated by ficoll gradient centrifugation. PBMCs were washed three times with DMEM and stained with tryptan blue, and live cells were counted on a hemocytometer and plated on top of breast cancer cells. In more detail, 10^6^ PBMCs were plated on top of (MCF-7 and MDA-MB-231, or TRAF3-expressing counterparts) adherent breast cancer cells (10^5^ cells) on a 12-well plate in a total volume of 2 mL and co-cultured with DMEM/10%FBS/pen-strep for 3 days. The culture medium was changed every second day. The day of the experiment, the supernatant was removed and centrifuged, the pellet consisting of PBMCs and cancer cells was analyzed for cell activation profile, and the cytokine concentration was determined in the supernatant. In order to avoid inter-donor heterogeneity, whole blood was obtained from a single healthy, multiparous, female (age 35–45) volunteer after informed consent, and the experiment was repeated twice from the same donor.

### 4.7. Flow Cytometry Analysis

PBMCs were resuspended in staining buffer (2% FBS in PBS) and incubated for 30 min at RT in the dark, with fluorochrome-conjugated antibodies for the respective markers expressed on the surface of the cells as follows: CD3 (cat#557832), CD4 (cat#555346), CD8 (cat#565310), CD16 (cat#561248), CD25 (cat#555434), CD25 (cat#555432), CD69 (cat#555531), HLA-DR (cat#562304), CD28 (cat#555728), CD38 (cat#555460), and CD127 (cat#560551) all from BD Biosciences (San Jose, CA, USA) and CD20 (cat#A07772) and CD56 (cat#A07788) from Beckman Coulter Inc. (Brea, CA, USA). In order to determine the live and dead cell populations, cells were treated according to the manufacturer kit procedure (Apoptosis/Necrosis Detection Kit, ENZ-51002, Enzo Life Sciences, Inc, Farmingdale, NY, USA). Samples were analyzed on a BD FACSCelesta Cell Analyzer (San Jose, CA, USA), and data were processed in FlowJo software, V10.10 (BD Life Sciences, Ashland, OR, USA).

### 4.8. Supernatant Cytokine Measurement

Cytokine levels (IFN-γ, TNF, IL-10, IL-6, IL-4, IL-2) were determined by flow cytometry. Cytokine concentration in supernatants from co-culture of PBMCs and breast cancer cells was measured using a cytometric bead array (CBA) assay (Human Th1/Th2 Cytokine Kit II, cat#551809, BD Biosciences, San Jose, CA, USA). The samples were analyzed on a BD FACSCelesta Cell Analyzer, and data were processed in FlowJo software, V10.10 (BD Life Sciences, Ashland, OR, USA).

### 4.9. Enrichment Analyses

To perform Gene Ontology (GO) enrichment, we performed over-representation analysis (ORA) using Metascape [22] for the 429 significant protein interactors from MS data and WebGestalt [23] for the *TRAF3* positively correlated, co-expressed genes (6306) from the TCGA BRCA dataset.

### 4.10. Statistical Analysis

Student’s *t*-test or Mann–Whitney U test was used for comparisons between groups. Differences in *TRAF3* expression between clinicopathological parameters of more than two categories were determined using the Kruskal–Wallis test. To account for pairwise comparisons, a Bonferroni correction was applied. All experimental data (other than MS, ORA and single-cell RNA seq data) were analyzed with the SPSS program SPSS^®^ release 23.0, Chicago, IL, USA). Any *p*-value < 0.05 was considered statistically significant (ns: *p* ≥ 0.05; ∗ *p* < 0.05; ∗∗ *p* < 0.01; ∗∗∗ *p* < 0.001).

### 4.11. Single-Cell RNA-Seq Dataset Analysis

Single-cell RNA sequencing (scRNA-seq) data were retrieved from a publicly available breast cancer single-cell atlas published by Wu et al. [24], comprising 26 primary human breast tumors, including 11 ER-positive, 5 HER2-positive, and 10 triple-negative breast cancers (TNBCs). Dimensionality reduction and cell type annotation (UMAP) were performed using Seurat v4 [49], following the clustering and major cell type labels provided by Wu et al. A total of 12,664 cancer epithelial (CE) cells were extracted for further analysis. *TRAF3* expression was quantified using log-normalized data. Differential expression (DE) analysis between *TRAF3*-positive and *TRAF3*-negative CE cells was performed using the limma R package [50]. The design matrix accounted for TRAF3 status, patient-of-origin, and the different CE subtypes (numeric encoding of the minor CE subtypes: Cancer Cycling, Cancer Her2 SC, Cancer LumB SC, Cancer Basal SC, Cancer LumA SC) to reduce confounding by inter-patient variability and subtype composition. The top DE genes with FDR < 0.05 and |log2FC| > 0.1 from *TRAF3*-positive versus *TRAF3*-negative CE cells were subjected to Gene Ontology (GO) enrichment analysis using the DAVID platform (Database for Annotation, Visualization, and Integrated Discovery) [51]. Annotation stringency was set to medium, and the analysis focused on Biological Process and Molecular Function GO terms (excluding Cellular Component terms). Functional clustering was applied, and the top 10 clusters based on enrichment score were selected for interpretation. Statistical analyses for scRNAseq data were conducted in R v4.2.

## Data Availability

Data from this work are available upon reasonable request.

## Supplementary Materials

The following supporting information can be downloaded at: https://www.mdpi.com/article/10.3390/ijms27104414/s1.
