# Supplementary material for: Dual Role of Cancer Epithelial-Specific TRAF3 in Regulating Breast Cancer Cell Survival and Lymphocyte Activity
Source: Int J Mol Sci. 2026 May 15;27(10):4414. doi: 10.3390/ijms27104414 (PMC13207503; doi:10.3390/ijms27104414)
Supplement: Supplementary file 1 [file ijms-27-04414-s001.zip › Supplementary Figure Legends.pdf]

Supplementary Figure Legends

Title: Dual role of cancer epithelial-specific TRAF3 in regulating breast cancer cell survival and lymphocyte activity.

Chaido Sirinian[a,*], Anne-Lise de Lastic[b], Harry Zaverdas[c], Martha Nifora[d], Dimitra Georgakopoulou[b], Martina Samiotaki[e], Maria Ioanna Argentou[f], Stavros Peroukidis[g], Søren E. Degn[h], Maria Rusan[i,j,k], Konstantinos Theofilatos[l,m], Seferina Mavroudi[c], Anastasios D. Papanastasiou[d,#] and Angelos Koutras [a,#]

Supplementary Figure S1. *TRAF3* mRNA expression analysis from GOBO breast cancer. (a) *TRAF3* mRNA expression association with OS in PAM50 divided group of tumors, where HER2 enriched group presents a statistically significant correlation with *TRAF3* expression. (b) *TRAF3* mRNA expression association with DMFS in PAM50 divided group of tumors. Both Basal and HER2 enriched group of tumors present a statistically significant correlation with *TRAF3* mRNA.

Supplementary Figure S2. TRAF3 in breast cancer cells affects cellular phenotype and protein expression. (a) Brightfield images of MCF-7 and MDA-MB-231 control and TRAF3-expressing cancer cells. MCF7-TRAF3 cells lose their cohesiveness and present a partial EMT-like reversion. (b) MDA-MB-231 control and TRAF3-expressing cells ICC stained for the indicated proteins. (c) BCL-2 protein expression presents a reverse correlation with *TRAF3* mRNA expression in the TCGA BRCA cohort. (d) Western blot for TRAF2, phospho-IkBa (p-IkBa) and phospho-p65 (p65) in MCF-7 and MDA-MB-231 cells.

Supplementary Figure S3. Full list of GO terms that were found significantly (adj. P-Value of enrichment <0.05) enriched in the top 100 (with the lowest adj p-value) genes identified via differential expression analysis between TRAF3+ and TRAF3- cancer epithelial (CE) cells. X-Axis represents the Fold Enrichment, and Y-Axis represents all the immune-related Biological Process and Molecular Function GO terms, grouped into clusters (Clr 10, Clr 2, etc.) based on functional similarity, along with the Enrichment Score of the cluster in parentheses. Dot size is analogous to the number of specific genes associated with each GO term, while their color gradient corresponds to the FDR-adjusted p-value (Q value). Abbreviations used: PR (Positive Regulation), R (Regulation), prd (production), MM (Molecular Mediator), MBP (Macromolecule Biosynthetic Process), MMP (Macromolecule Metabolic Process).

Supplementary Figure S4. TRAF3 and control cancer cells co-cultured with PBMCs and FACS analysis. (a) Brightfield images of MCF-7 and MDA-MB-231 co-cultured with PBMCs. Arrowheads depict cancer cell/PBMC aggregates. (b) Gating strategy for T-regulatory cell subsets FACs analysis from MCF-7/PBMC control and TRAF3-expressing cells and MDA-MB-231/PBMCs control and TRAF3 expressing cells. (c) Gating strategy for NK cell subsets FACS analysis from MCF-7/PBMC control and TRAF3-expressing cells and MDA-MB-231/PBMCs control and TRAF3 expressing cells.

Supplementary Figure S5. (a) FACS analysis for dead/alive cancer cell populations from MDA231-control and MDA231-TRAF3 cells co-cultured with PBMCs. TRAF3 seems to affect dead/alive cell ratios in the PBMC co-culture system, making cancer cells more sensitive to the action of PBMCs. (b) FACS analysis for dead/alive cancer cell ratios in the MCF7-TRAF3 alone culture, depicting the effect of TRAF3 in death/apoptosis on MCF-7 cells. (c) PD-L1 ICC on MDA231-control and MDA231-TRAF3 cells. Arrowheads depict PD-L1 positive cells. (d) CD274 mRNA expression from the cancer epithelial cell compartment in the TRAF3-positive and negative cells (single cell data), presenting with a significant negative correlation. (e) PD-L1 (CD274) protein expression is negatively correlated with TRAF3 protein expression in 22 breast cancer cell lines from the Cancer Cell Line Encyclopedia (Broad, 2019).
