# Supplementary material for: Dual Role of Cancer Epithelial-Specific TRAF3 in Regulating Breast Cancer Cell Survival and Lymphocyte Activity
Source: Int J Mol Sci. 2026 May 15;27(10):4414. doi: 10.3390/ijms27104414 (PMC13207503; doi:10.3390/ijms27104414)
Supplement: Supplementary file 1 [file ijms-27-04414-s001.zip › Supplementary Materials and Methods.pdf]

Supplementary Materials and Methods

Title: Dual role of cancer epithelial-specific TRAF3 in regulating breast cancer cell survival and lymphocyte activity.

Chaido Sirinian[a,*], Anne-Lise de Lastic[b], Harry Zaverdas[c], Martha Nifora[d], Dimitra Georgakopoulou[b], Martina Samiotaki[e], Maria Ioanna Argentou[f], Stavros Peroukidis[g], Søren E. Degn[h], Maria Rusan[i,j,k], Konstantinos Theofilatos[l,m], Seferina Mavroudi[c], Anastasios D. Papanastasiou[d,#] and Angelos Koutras [a,#]

mRNA expression data sets analyses

For the TCGA cohort 960 patients/cases with complete data were employed for analysis of TRAF3 expression (RNAseq V2 RSEM) with the relevant clinicopathological data. GOBO (Gene expression-based Outcome for Breast cancer Online) employed 1881 tumor samples of which: ER+ tumors 1225, ER- tumors 395, Untreated tumors 927, TAM treated tumors 326. From the Cancer Cell Line Encyclopedia (Broad, 2019) 22 breast cancer cell lines were employed through cBioPortal (AU565, BT20, BT549, CAL120, CAL51, CAL851, CAMA1, EFM192A, HCC1143, HCC1395, HCC1806, HCC1937, HCC1954, HCC70, JIMT1, KPL1, MCF7, MDAMB157, MDAMB231, MDAMB453, MDAMB468, T47D).

Immunoblotting and Immunocytochemistry

For western blot analysis cells were lysed in 0.5%NP-40 lysis buffer (150mM NaCl, 20mM HEPES, 0.5mM EDTA, 1mM Na_3VO_4, proteinase inhibitor cocktail (Calbiochem)). Protein lysates were analyzed in 10% SDS-PAGE electrophoresis and transferred onto polyvinylidene difluoride membrane (Millipore) following incubation with the appropriate antibodies overnight at 4°C. Next day membranes were incubated for 1h at RT with a horseradish peroxidase-conjugated secondary antibody (anti-goat;AP180P,1:3000,Millipore, anti-mouse;AP182P, 1:3000, Millipore and anti-rabbit; #7076, 1:3000, CST). All initial images were transformed into grayscale for clarity.

For Immunocytochemistry (ICC) adherent cells were cultured on coverslips. Cells were fixed on 4% paraformaldehyde for 15 min and cells were permeabilized with 0,3% Triton. Non-specific binding was inhibited by treating cells with 1% bovine serum albumin (BSA) for 30 min. Cells were incubated overnight at 4°C with the primary antibody followed by the Dako REAL™

EnVision^{TM} Detection System (Dako) for 30min. Immunoreactions were visualized by the application of 3,3'-diaminobenzidine (DAB). All slides were counterstained with hematoxylin and mounted.

Single-cell RNA-seq Dataset Analysis

In total, 130,246 single cells were profiled, capturing malignant epithelial cells alongside diverse immune, stromal, and endothelial populations. The raw count matrices for these 130,246 cells were filtered based on standard quality-control metrics, retaining cells with 200–5,000 detected genes and ≤10% mitochondrial transcript content, resulting in 81,389 high-quality cells for downstream analysis.

TRAF3 expression was quantified using log-normalized data, and cells were classified as TRAF3-positive (TRAF3+; expression >0, 764 cells) or TRAF3-negative (TRAF3-; expression =0, 11,900 cells). Violin plots depicting TRAF3 expression distributions in each category were generated, and the statistical significance of TRAF3 expression in each category compared to the expression in all other categories combined was assessed using Wilcoxon rank-sum tests, and p-values were adjusted with the Benjamini–Hochberg (BH) method. Significance was annotated on plots with asterisks (*) for adjusted $p < 0.05$.

Linear models were fitted for each gene, followed by empirical Bayes moderation (eBayes) and multiple-testing adjustment using the BH method. The top 100 genes with the lowest adjusted p-values were used for downstream enrichment analysis. For visualization of DE patterns, volcano plots were constructed and immunologically relevant genes were categorized as: Immunogenicity: Immunogenicity/Antigen Presentation, MHC-I: MHC class I pathway (CD8$^+$ T-cell recognition), MHC-II: MHC class II (tumor-intrinsic or APC-mediated), Checkpoint: Checkpoint blockade / immune modulation, Infiltration: Increase immune infiltration into tumors, Non-self: Promote tumor cell recognition as "non-self", while selected genes not belonging to the above categories were labeled as "Other."
