# Supplementary material for: Dual Role of Cancer Epithelial-Specific TRAF3 in Regulating Breast Cancer Cell Survival and Lymphocyte Activity
Source: Int J Mol Sci. 2026 May 15;27(10):4414. doi: 10.3390/ijms27104414 (PMC13207503; doi:10.3390/ijms27104414)
Supplement: Supplementary file 1 [file ijms-27-04414-s001.zip › Supplementary Table Legends.pdf]

Supplementary Table Legends

Title: Dual role of cancer epithelial-specific TRAF3 in regulating breast cancer cell survival and lymphocyte activity.

Chaido Sirinian[a,*], Anne-Lise de Lastic[b], Harry Zaverdas[c], Martha Nifora[d], Dimitra Georgakopoulou[b], Martina Samiotaki[e], Maria Ioanna Argentou[f], Stavros Peroukidis[g], Søren E. Degn[h], Maria Rusan[i,j,k], Konstantinos Theofilatos[l,m], Seferina Mavroudi[c], Anastasios D. Papanastasiou[d,#] and Angelos Koutras [a,#]

Supplementary Table S1.

Cases from the TCGA BRCA cohort, with WSI, employed for TIL scoring. The table presents patient /sample ID, *TRAF3* mRNA expression and TIL scores.

Supplementary Table S2.

Detailed results of FACS analyses of TRAF3-expressing and control cancer cells, co-cultured with PBMCs.

Supplementary Table S3.

Detailed results of the whole panel of cytokines employed in this work [BD Cytometric Bead Array (CBA) Human Th1/ Th2 Cytokines Kit II, cat#551809].
