# Supplementary material for: Dual Role of Cancer Epithelial-Specific TRAF3 in Regulating Breast Cancer Cell Survival and Lymphocyte Activity
Source: Int J Mol Sci. 2026 May 15;27(10):4414. doi: 10.3390/ijms27104414 (PMC13207503; doi:10.3390/ijms27104414)
Supplement: Supplementary file 1 [file ijms-27-04414-s001.zip › Sup. Figure S1.pptx]

## Slide 1
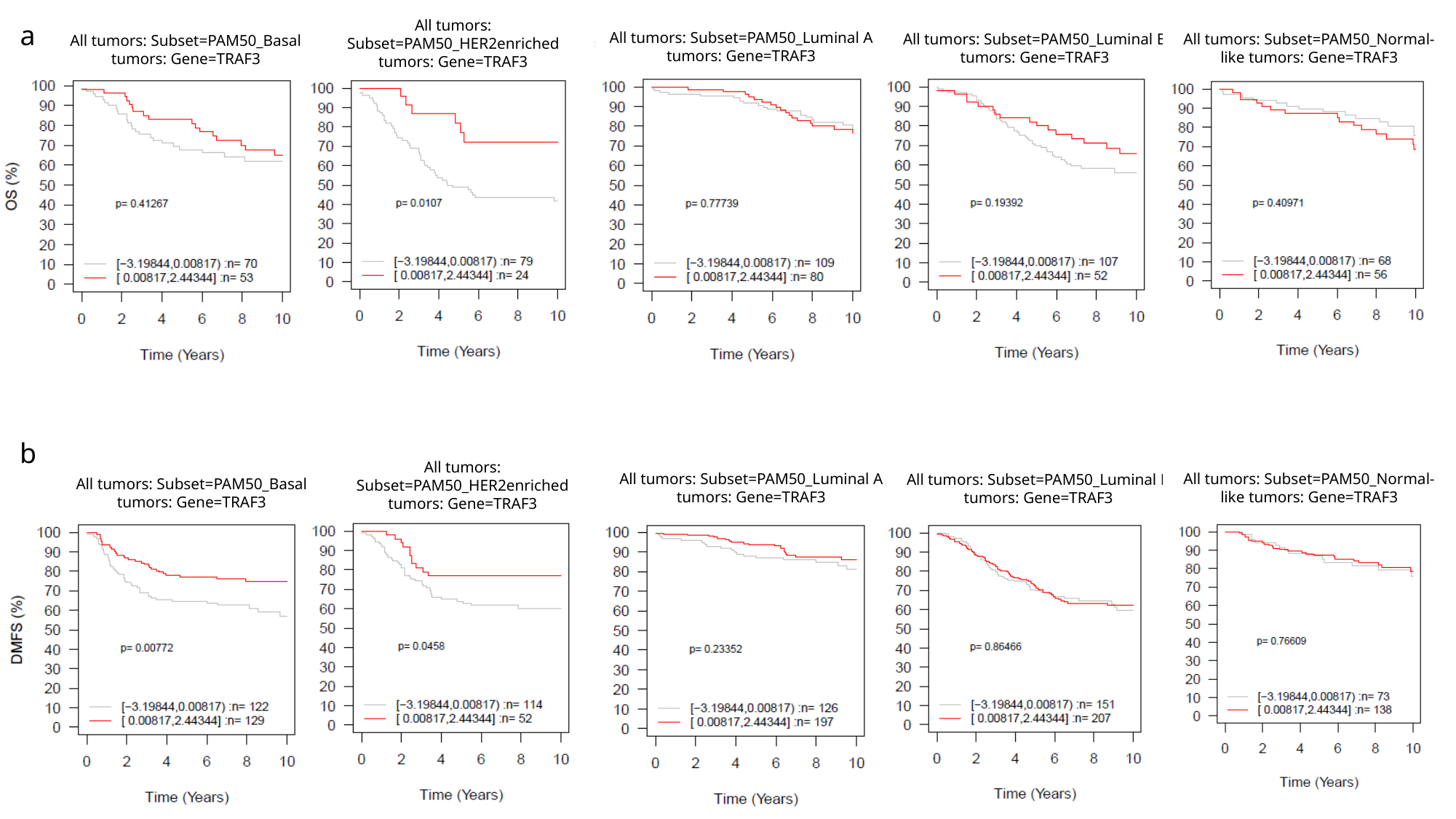

All tumors: Subset=PAM50_HER2enriched tumors: Gene=TRAF3
a
All tumors: Subset=PAM50_Luminal A tumors: Gene=TRAF3
All tumors: Subset=PAM50_Luminal B tumors: Gene=TRAF3
All tumors: Subset=PAM50_Normal-like tumors: Gene=TRAF3
All tumors: Subset=PAM50_Basal tumors: Gene=TRAF3
b
All tumors: Subset=PAM50_HER2enriched tumors: Gene=TRAF3
All tumors: Subset=PAM50_Luminal A tumors: Gene=TRAF3
All tumors: Subset=PAM50_Normal-like tumors: Gene=TRAF3
All tumors: Subset=PAM50_Luminal B tumors: Gene=TRAF3
All tumors: Subset=PAM50_Basal tumors: Gene=TRAF3
