# Supplementary material for: Dual Role of Cancer Epithelial-Specific TRAF3 in Regulating Breast Cancer Cell Survival and Lymphocyte Activity
Source: Int J Mol Sci. 2026 May 15;27(10):4414. doi: 10.3390/ijms27104414 (PMC13207503; doi:10.3390/ijms27104414)
Supplement: Supplementary file 1 [file ijms-27-04414-s001.zip › Sup. Figure S2 Rev.pptx]

## Slide 1
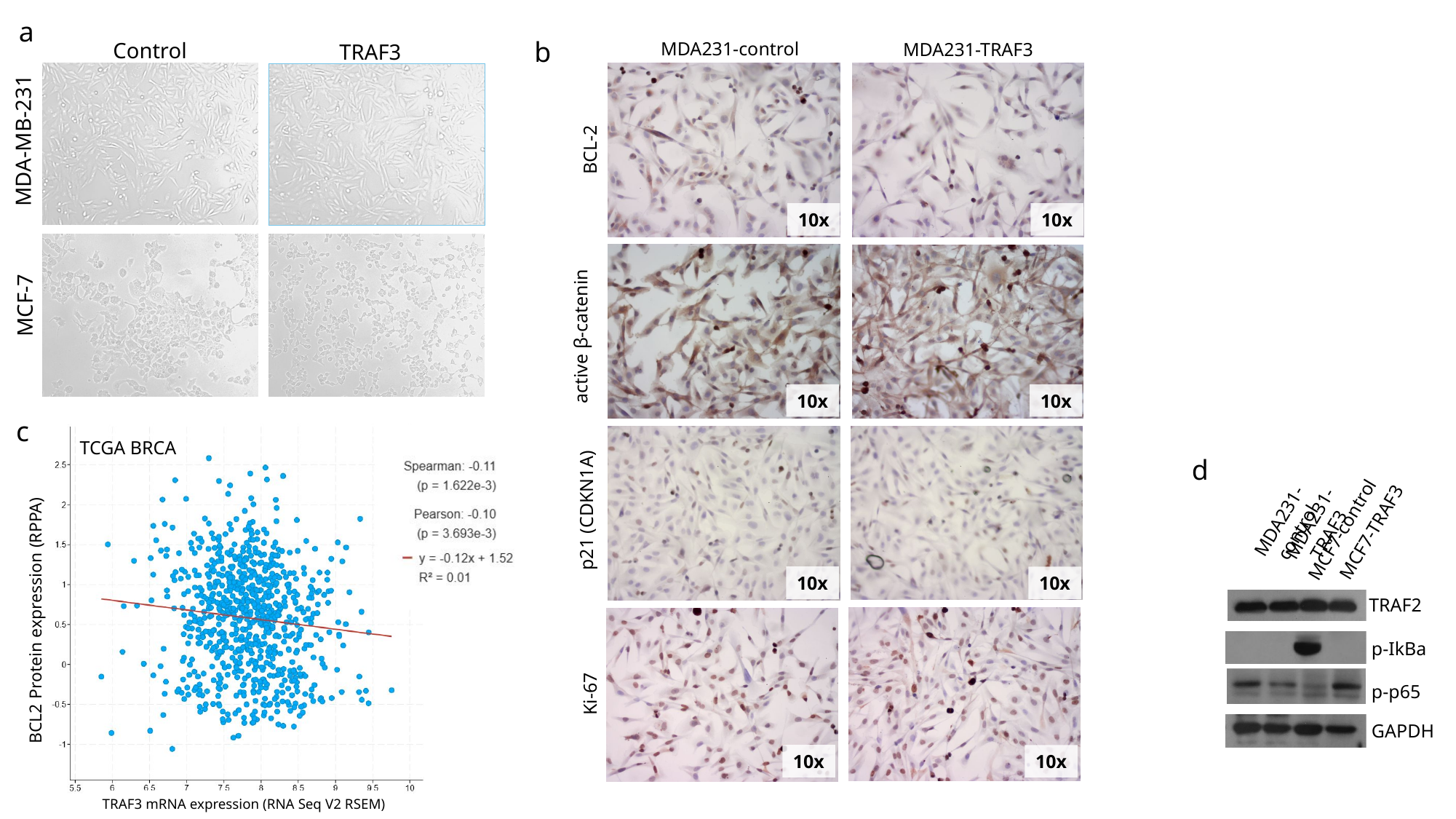

a
Control
TRAF3
MDA-MB-231
MCF-7
b
MDA231-control
MDA231-TRAF3
BCL-2
10x
10x
active β-catenin
10x
10x
c
TCGA BRCA
d
p21 (CDKN1A)
MDA231-control
MDA231-TRAF3
MCF7-control
MCF7-TRAF3
10x
10x
TRAF2
BCL2 Protein expression (RPPA)
p-IkBa
p-p65
Ki-67
GAPDH
10x
10x
TRAF3 mRNA expression (RNA Seq V2 RSEM)
