# Supplementary material for: Dual Role of Cancer Epithelial-Specific TRAF3 in Regulating Breast Cancer Cell Survival and Lymphocyte Activity
Source: Int J Mol Sci. 2026 May 15;27(10):4414. doi: 10.3390/ijms27104414 (PMC13207503; doi:10.3390/ijms27104414)
Supplement: Supplementary file 1 [file ijms-27-04414-s001.zip › Sup. Figure S4.pptx]

## Slide 1
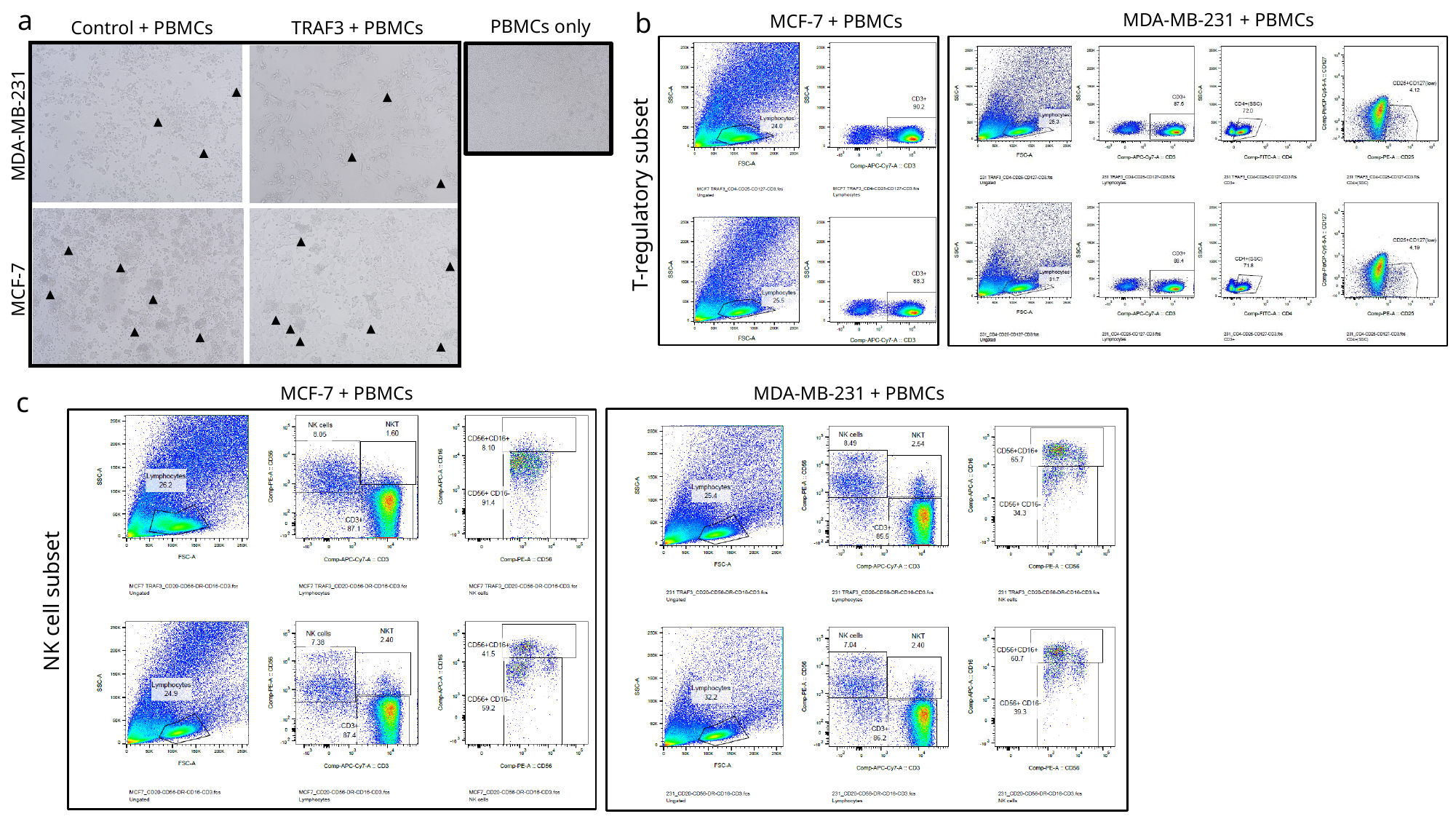

a
b
MDA-MB-231 + PBMCs
MCF-7 + PBMCs
PBMCs only
Control + PBMCs
TRAF3 + PBMCs
MDA-MB-231
T-regulatory subset
MCF-7
MDA-MB-231 + PBMCs
MCF-7 + PBMCs
c
NK cell subset
