# Supplementary material for: Dual Role of Cancer Epithelial-Specific TRAF3 in Regulating Breast Cancer Cell Survival and Lymphocyte Activity
Source: Int J Mol Sci. 2026 May 15;27(10):4414. doi: 10.3390/ijms27104414 (PMC13207503; doi:10.3390/ijms27104414)
Supplement: Supplementary file 1 [file ijms-27-04414-s001.zip › Sup. Figure S5.pptx]

## Slide 1
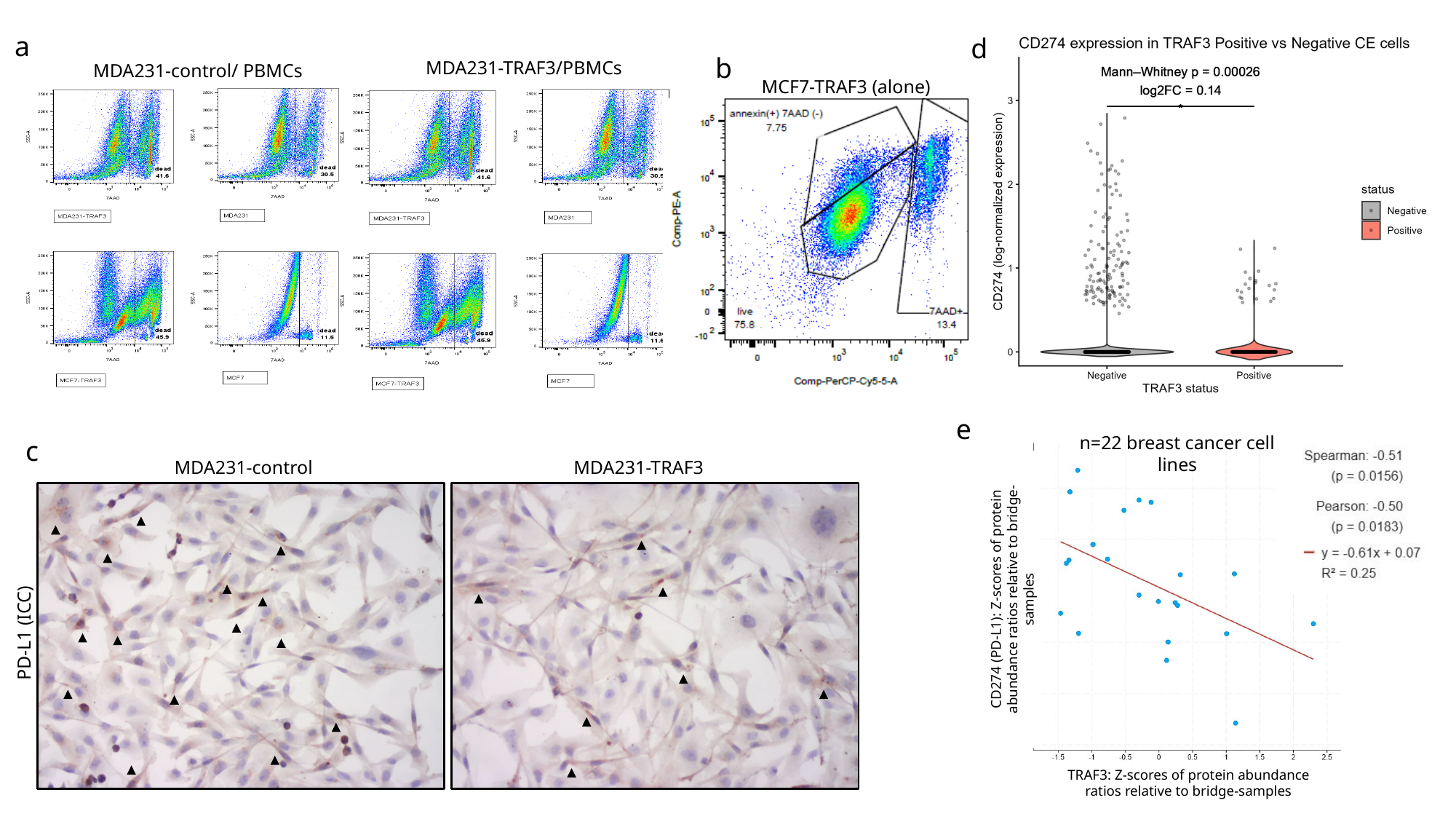

a
d
b
MDA231-TRAF3/PBMCs
MDA231-control/ PBMCs
MCF7-TRAF3 (alone)
e
CD274 (PD-L1): Z-scores of protein abundance ratios relative to bridge-samples
TRAF3: Z-scores of protein abundance ratios relative to bridge-samples
n=22 breast cancer cell lines
c
MDA231-TRAF3
MDA231-control
PD-L1 (ICC)
