# Supplementary figures and images for: Dual Role of Cancer Epithelial-Specific TRAF3 in Regulating Breast Cancer Cell Survival and Lymphocyte Activity
Source: Int J Mol Sci. 2026 May 15;27(10):4414. doi: 10.3390/ijms27104414 (PMC13207503; doi:10.3390/ijms27104414)

## Slide 1
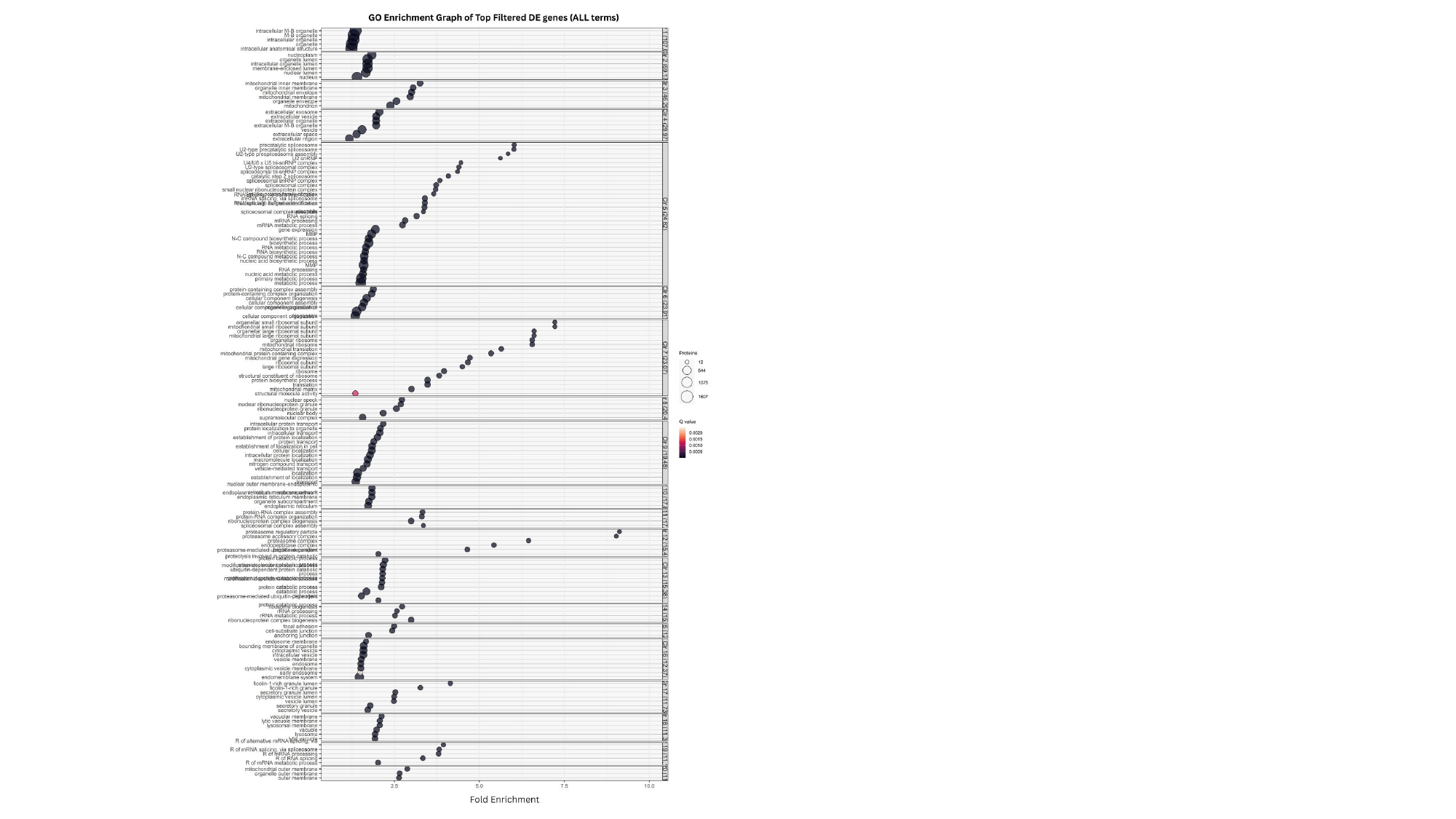

Supplement: Supplementary file 1 [file ijms-27-04414-s001.zip › Sup. Figure S3 Rev.pptx]
